# Skeletal Muscle Dysfunction and Exercise Intolerance in COPD and Idiopathic Pulmonary Fibrosis: Extracellular Vesicles as Candidate Mediators of a Lung–Muscle Axis

**DOI:** 10.3390/muscles5030055

**Published:** 2026-08-03

**Authors:** Georgios I. Barkas, Zoe Daniil, Ourania S. Kotsiou

**Affiliations:** 1 Laboratory of Human Pathophysiology, Department of Nursing, School of Health Sciences, University of Thessaly, 41500 Larissa, Greece; gbarkas-m@uth.gr; 2 Department of Respiratory Medicine, Faculty of Medicine, School of Health Sciences, University of Thessaly, 41110 Larissa, Greece

**Keywords:** extracellular vesicles, small extracellular vesicles, COPD, idiopathic pulmonary fibrosis, skeletal muscle dysfunction, muscle wasting, exercise intolerance, inter-organ communication

## Abstract

Skeletal muscle dysfunction and exercise intolerance are major extrapulmonary manifestations of chronic obstructive pulmonary disease (COPD) and idiopathic pulmonary fibrosis (IPF), yet their severity is not fully predicted by pulmonary impairment. This narrative review examines extracellular vesicles (EVs) as candidate mediators of lung–muscle communication within a broader network of inflammatory, metabolic, vascular, nutritional, and inactivity-related mechanisms. The evidence is asymmetrical. COPD provides direct human skeletal muscle evidence for quadriceps microRNA dysregulation, impaired protein synthesis and mitochondrial function, oxidative stress, and abnormalities of the regenerative microvascular niche; however, none of these observations demonstrates delivery of pathogenic cargo from the lung by EVs. In IPF, EV-mediated epithelial–mesenchymal signalling, fibroblast activation, and profibrotic remodelling are well supported within the lung, whereas skeletal muscle effects remain indirect. Accordingly, the lung–muscle EV axis should be viewed as a biologically plausible, evidence-weighted hypothesis rather than an established causal pathway. Progress will require experiments that identify the cellular source of EVs, trace their vascular transit and skeletal muscle uptake, and demonstrate functional cargo transfer using EV-depletion, rescue, and integrated muscle readouts. Conventional size and morphology measurements do not reliably distinguish muscle- from lung-derived EVs; source discrimination currently depends more on molecular cargo and cell-associated markers. Hypoxia and transient or sustained oxygen desaturation may modify EV release and cargo through HIF- and redox-sensitive signalling, but disease-specific evidence connecting these changes to lung-to-muscle transfer in COPD or IPF remains limited.

## 1. Introduction

Chronic respiratory diseases, particularly COPD and IPF, are defined anatomically by pulmonary pathology but clinically by their systemic consequences. Skeletal muscle wasting, weakness, and exercise intolerance are major determinants of disability, health-related quality of life, healthcare utilisation, and prognosis [1,2,3,4,5]. Locomotor muscle dysfunction is not explained solely by the severity of pulmonary impairment: patients with comparable airflow limitation or radiological fibrotic burden may differ substantially in quadriceps strength, oxidative capacity, fatigue resistance, physical activity, and functional status [2,3,4].

Exercise intolerance and skeletal muscle wasting are related but not interchangeable. Exercise capacity may be reduced without measurable muscle loss because ventilatory mechanics, dynamic hyperinflation, hypoxaemia, pulmonary vascular disease, cardiac comorbidity, anaemia, and deconditioning also contribute [3,6]. Conversely, muscle loss may occur as part of systemic cachexia or reflect nutritional vulnerability, inactivity, inflammation, oxidative stress, and altered protein turnover [1,7]. Involvement is regionally heterogeneous: lower-limb locomotor muscles, especially the quadriceps, are commonly affected in COPD, whereas the diaphragm may show distinct loading-related adaptations [3,6]. Quadriceps weakness and low muscle mass are also associated with exercise capacity and prognosis in IPF, although their mechanisms are less well characterised [4,5].

Because no integrated COPD or IPF study has demonstrated lung-origin EV release, vascular transit, skeletal muscle uptake, and functional cargo delivery, the proposed lung–muscle EV axis is treated here as a testable hypothesis rather than an established causal pathway [8,9,10].

The inflammatory spillover model remains relevant: mediators generated in diseased lungs may enter the circulation and contribute to catabolism. It is unlikely, however, to operate in isolation. Muscle dysfunction in COPD and IPF probably reflects interacting effects of systemic inflammation, hypoxaemia, oxidative stress, inactivity, nutritional imbalance, altered protein turnover, mitochondrial dysfunction, vascular injury, and impaired regeneration [1,2,3,4,6,7]. EVs provide a complementary—not preferential—mechanism because they can carry protected proteins, lipids, and regulatory RNAs between cells [8,11,12]. Whether pulmonary EVs contribute materially to distal muscle pathology remains unresolved.

MicroRNAs are discussed because some of the strongest COPD muscle data concern regulatory RNA networks and because microRNAs can be transported by EVs. The evidentiary distinction is essential: miR-542-3p/-5p dysregulation has been demonstrated in COPD quadriceps, whereas miR-23a has anti-atrophy activity and can undergo exosome-mediated export in muscle-atrophy models [13,14,15]. Neither observation proves that these microRNAs originate in the lung or are delivered to skeletal muscle by EVs. The IPF evidence base is different, being strongest for intrapulmonary EV-mediated epithelial reprogramming, fibroblast activation, and profibrotic signalling [10,16,17,18].

The added value of this review is therefore not another catalogue of pulmonary EV findings. It develops an evidence-weighted lung–muscle framework that separates direct human muscle observations, pulmonary EV biology, indirect vascular or systemic evidence, and hypothesis-generating cross-organ mechanisms. It also identifies the experimental threshold required to establish causality and evaluates therapeutic translation without conflating pulmonary feasibility with treatment of respiratory disease-associated muscle dysfunction.

## 2. MISEV-Aligned EV Terminology and Methodological Cautions

EV terminology follows MISEV-aligned principles [8]. The generic term extracellular vesicles is used unless vesicle biogenesis has been experimentally demonstrated. Small EVs is preferred for operationally isolated nanoscale vesicles when endosomal origin has not been established. The term exosome is retained only when referring to studies that explicitly used that term, engineered exosome platforms, or biological contexts in which endosomal biogenesis is central to the original experimental claim [8].

This distinction is mechanistically important. COPD and IPF studies use heterogeneous isolation and characterisation approaches, including differential ultracentrifugation, precipitation, size-exclusion chromatography, immunoaffinity capture, flow cytometry, nanoparticle tracking analysis, electron microscopy, and proteomic or RNA profiling [8,9,19,20]. These methods enrich overlapping but non-identical vesicle populations; therefore, exosome-specific conclusions should not be inferred from bulk EV preparations without appropriate evidence of size, density, morphology, markers, and biogenesis [8].

Conventional EV morphology provides limited source discrimination. Lung- and muscle-derived preparations both contain membrane-bound particles with substantially overlapping size distributions, and their apparent diameter and shape vary with biogenesis, isolation, fixation, imaging, and the degree of enrichment for specific EV subpopulations [8,17,20,21]. No validated particle morphology uniquely identifies pulmonary or skeletal muscle origin. Consequently, morphology should be combined with molecular cargo, source-associated surface markers, and orthogonal source-validation methods rather than used alone to infer tissue origin.

For a proposed lung–muscle axis, source attribution is a major limitation. Bulk plasma EV preparations cannot be assumed to be lung-derived, and pre-analytical handling, storage, normalisation, and marker selection can materially alter apparent abundance and cargo. Bronchoalveolar lavage (BAL)-derived EVs may better reflect local pulmonary biology, whereas circulating EVs represent composite populations that may include contributions from pulmonary, endothelial, immune, platelet, adipose, and skeletal muscle sources. Claims of lung-origin EV effects on muscle therefore require source-specific markers, genetic labelling or lineage tracing, single-vesicle phenotyping where feasible, and functional uptake assays in relevant recipient cells.

## 3. Disease-Specific EV Evidence in COPD and IPF: Pulmonary Signals, Circulating EVs, and Muscle-Relevant Findings

### 3.1. Established Skeletal Muscle Features in COPD

Skeletal muscle dysfunction is an established systemic manifestation of COPD and is clinically distinct from loss of lung function. It most consistently affects the quadriceps, where reduced strength, endurance, and muscle mass contribute independently to impaired exercise capacity, poorer health status, healthcare use, and mortality [1,2,3,6]. The phenotype is heterogeneous: some patients develop low fat-free mass or cachexia, whereas others retain muscle mass but exhibit substantial weakness, indicating that muscle quantity and contractile quality should be assessed separately [1,2,3,6,7]. Lower-limb involvement is generally more pronounced than upper-limb involvement, consistent with the combined effects of deconditioning, reduced habitual loading, and disease-related biological injury [2,3,22]. Recent synthesis further frames sarcopenia as a measurable and potentially treatable COPD trait [23].

At tissue level, COPD quadriceps commonly shows a shift from oxidative type I fibres toward more glycolytic type II fibres, reduced capillary density, lower oxidative-enzyme activity and mitochondrial capacity, and earlier lactate accumulation and fatigability during exercise [2,3,22,24,25]. These abnormalities coexist with oxidative and nitrosative stress, systemic and intramuscular inflammatory signalling, impaired anabolic responses, activation of ubiquitin–proteasome and autophagy-related catabolic pathways, and dysregulated protein synthesis [2,6,7,14,24,26,27]. Satellite-cell dysfunction and disruption of the regenerative microvascular niche provide an additional layer: COPD-derived precursor cells show impaired myogenic behaviour in disease-relevant environments, cultured satellite cells retain atrophic and oxidative-stress features, and human biopsy studies demonstrate increased capillary-to-satellite-cell distance [28,29,30,31]. Hypoxaemia, corticosteroid exposure, smoking, exacerbations, nutritional depletion, comorbidities, and physical inactivity may amplify these processes, but none is sufficient alone to explain the full phenotype [1,2,3,6,7]. A 2026 perspective also identifies glucocorticoid-associated weakness during exacerbations as a clinically relevant and potentially modifiable contributor [32].

These established observations define the muscle phenotype that any proposed lung–muscle mediator must explain. Importantly, the evidence supports a multifactorial model rather than a single circulating mechanism: pulmonary limitation constrains activity, while systemic inflammation, redox imbalance, vascular dysfunction, altered regeneration, mitochondrial impairment, and disturbed protein turnover interact within skeletal muscle [1,2,3,6,7].

### 3.2. Extracellular Vesicles and Muscle Wasting in COPD

Evidence implicating EVs specifically in COPD-associated muscle wasting remains limited and should be separated into three levels. First, direct human muscle evidence demonstrates EV-relevant cargo abnormalities without establishing vesicular delivery: miR-542-3p and miR-542-5p are increased in COPD quadriceps and suppress ribosomal and protein-synthesis pathways [14], while miR-23a restrains MuRF-1 and Atrogin-1 expression and can be depleted from atrophying muscle through exosome-mediated export in experimental models [13,15]. These findings connect regulatory RNAs, proteostasis, and EV biology, but they neither identify a pulmonary source nor show transfer into COPD muscle.

Second, COPD alters pulmonary, bronchoalveolar-lavage, epithelial, endothelial, and circulating EV populations [9,11,19,20,33]. BAL-derived EVs show disease-associated protein and microRNA signatures, although circulating EV profiles do not simply reproduce BAL changes [19,20]. Recent source-defined experiments further show that EVs from primary COPD airway epithelial cells can be internalised by endothelial cells and promote inflammation, apoptosis, monocyte adhesion, and atherosclerotic injury through reduced EV miR-141-3p and activation of the PDCD4/NF-κB pathway [34]. This provides proof that COPD airway-cell EVs can exert extrapulmonary vascular effects in experimental systems. It remains indirect for muscle wasting, but is relevant because endothelial dysfunction, impaired perfusion, and disturbance of the capillary–satellite-cell niche could plausibly aggravate muscle hypoxia and regeneration [29,35,36].

Third, preclinical EV studies outside COPD show that vesicular cargo can modify muscle catabolism and regeneration. Mesenchymal-stromal-cell EVs attenuate experimental atrophy through miR-486-5p/FoxO1 and miR-145-5p/activin-receptor pathways, while hypoxia-conditioned EVs can enhance satellite-cell activation through miR-210-3p/KLF7 signalling [37,38,39]. These studies demonstrate mechanistic feasibility, not disease-specific causality. No study has yet shown the complete sequence required to establish an EV-mediated COPD lung–muscle axis: release from a defined diseased pulmonary cell, vascular transit, skeletal-muscle uptake, functional cargo delivery, induction of atrophy or weakness, and reversal by EV depletion or cargo-specific rescue. EVs should therefore be presented as candidate contributors operating alongside established catabolic, metabolic, vascular, nutritional, and inactivity-related mechanisms rather than as proven drivers of COPD muscle wasting.

### 3.3. IPF: Pulmonary EV Biology with Indirect Muscle Implications

In IPF, the most robust EV evidence is intrapulmonary. EVs participate in epithelial–mesenchymal communication, fibroblast activation, extracellular matrix remodelling, and profibrotic signalling [10,16,17,18]. Mechanistic examples include inhibition or dysregulation of TGF-β/WNT crosstalk by bronchial epithelial cell-derived EVs, syndecan-1-dependent epithelial reprogramming, and fibroblast-derived EV cargo containing SFRP1 [16,17,18]. These studies provide molecular evidence for EV-mediated disease amplification or modulation within fibrotic lung tissue.

Direct skeletal muscle consequences remain untested. Exercise intolerance, dyspnoea, hypoxaemia, inactivity, nutritional vulnerability, and systemic illness can impair muscle in IPF independently of EV trafficking [4,5]. Current data do not show that IPF-derived EVs enter the circulation, reach skeletal muscle, deliver functional cargo, or alter myofibres, satellite cells, endothelial cells, or fibro-adipogenic progenitors. IPF should therefore be regarded as a disease with strong pulmonary EV biology and a plausible systemic question—not established EV-mediated lung–muscle causality.

### 3.4. Morphological and Molecular Differences Between Lung- and Muscle-Derived EVs

Current evidence does not support a categorical morphology-based distinction between muscle-derived and lung-derived EVs. Both sources generate heterogeneous membrane-bound vesicles, including small EV-enriched populations with overlapping nanoscale dimensions. Differences reported between studies are more plausibly explained by source-cell state, vesicle biogenesis, disease context, and analytical workflow than by a stable organ-specific shape or diameter [8,17,20,21]. Thus, conventional electron microscopy or particle-size analysis cannot by itself assign circulating EVs to the lung or skeletal muscle.

Molecular composition is more informative, although no single marker is perfectly tissue-exclusive. Skeletal muscle-derived EV populations have been associated with alpha-sarcoglycan (SGCA), muscle-enriched microRNAs such as miR-1, miR-133a/b, and miR-206, and proteins linked to contraction, energy metabolism, myogenesis, and proteostasis [21,40,41]. Experimental evidence also suggests that many skeletal muscle EVs accumulate within the muscle interstitium and exert local paracrine effects, cautioning against the assumption that muscle necessarily contributes a large endocrine EV flux to plasma [41]. Electrical-pulse stimulation of human myotubes changed EV microRNA and protein cargo without a parallel change in vesicle size or number, further indicating that functional state may be reflected more strongly in cargo than in morphology [21].

Pulmonary EV cargo is similarly source- and context-dependent. COPD bronchoalveolar-lavage EVs show altered protein and microRNA signatures [20], while EVs released by primary COPD airway epithelial cells contain reduced miR-141-3p and can promote endothelial inflammation, apoptosis, monocyte adhesion, and atherosclerotic change through the PDCD4/NF-kappaB axis [34]. In fibrotic lung models, fibroblast-derived EVs contain SFRP1 and other signals implicated in WNT-related profibrotic communication [17]. These findings suggest that epithelial, endothelial, immune, and fibroblast EV cargo may provide more biologically meaningful source information than size or shape; however, available markers remain insufficiently validated for unambiguous source assignment in mixed plasma samples.

A major limitation is the absence of paired, source-resolved human studies that directly compare lung- and muscle-derived EV morphology and molecular cargo in the same patients with COPD or IPF. No study has yet mapped these features across stable disease, acute exacerbation, progressive fibrosis, pulmonary rehabilitation, or different severities of resting, exertional, nocturnal, or transient desaturation. Plasma EV profiles also integrate age, smoking, renal and cardiovascular comorbidity, medication use, platelet activation, exercise, and muscle mass. Accordingly, the proposed source-specific distinctions remain provisional and should be interpreted as candidate signatures rather than validated diagnostic or mechanistic classifiers.

## 4. Unresolved Trafficking and Uptake Barriers

The central question is whether EVs released from diseased lung tissue reach skeletal muscle in sufficient quantity, retain biologically active cargo, and engage the appropriate recipient cells. Broader EV research provides biological precedents but not direct COPD/IPF proof. In cancer models, EV surface integrins influence organotropic uptake [42]; animal biodistribution studies show frequent accumulation in liver, spleen, lung, and macrophage-rich compartments [43]; and endothelial transcytosis can permit EV passage across vascular barriers in context-dependent experimental systems [44,45].

At least six barriers must be resolved. First, pulmonary EVs must enter the circulation and traverse endothelial interfaces. Second, the endothelial glycocalyx may facilitate binding or impede passage. Third, integrins, tetraspanins, glycans, and other surface molecules may alter tropism, but no skeletal-muscle-specific targeting signature has been established for lung-derived EVs. Fourth, monocytes, macrophages, liver Kupffer cells, and splenic macrophages may intercept circulating EVs; endothelial cells are a separate vascular barrier or transit compartment, not an immune-cell subtype [43,46]. Fifth, plasma EVs are composite populations whose apparent cargo may reflect pulmonary injury, vascular injury, platelet activation, systemic inflammation, adipose tissue, or muscle itself. Sixth, no human study has integrated source labelling, vascular transit, endothelial crossing, skeletal muscle uptake, and functional cargo activity in one COPD or IPF model.

These limitations define the evidentiary threshold for causality. Future work should combine source-labelled EV systems, standardised isolation and characterisation, single-vesicle phenotyping, endothelial barrier models, EV-depletion and rescue experiments, recipient-cell uptake assays, and in vivo muscle outcomes. Precedents from cancer, drug-delivery, blood–brain-barrier, or animal biodistribution studies should be cited as precedents rather than presented as chronic respiratory disease evidence.

## 5. Skeletal Muscle Targets and Molecular Pathways

In COPD- and IPF-associated skeletal muscle dysfunction, weakness and atrophy may result from interacting disturbances in protein turnover, bioenergetics, oxidative stress, inflammation, hypoxaemia, inactivity, nutrition, vascular supply, and regeneration [1,2,3,5,7]. These processes identify plausible recipient compartments for EV-mediated or EV-modified signalling but are not, by themselves, evidence of pulmonary EV causality.

### 5.1. Myofibres and Protein Turnover

Myofibre loss reflects an imbalance between protein synthesis and degradation involving ubiquitin-proteasome and autophagy-lysosome pathways, impaired PI3K/Akt/mTOR signalling, and chronic inflammatory or oxidative stress [1,2,7,29,30]. In COPD quadriceps, miR-542-3p/-5p dysregulation is associated with reduced ribosomal function and protein synthesis [14]. This is direct COPD muscle evidence, but it does not identify the lung as the source or EVs as the delivery vehicle.

miR-23a provides a different level of evidence. It suppresses the atrophy-related ligases MuRF-1 and Atrogin-1, and experimental muscle atrophy can reduce intracellular miR-23a partly through exosome-mediated export [13,15]. The finding supports an EV-linked muscle mechanism within the experimental system studied; it should not be extrapolated to lung-derived cargo or to COPD/IPF without source, carriage, uptake, and rescue data.

### 5.2. Mitochondrial Dysfunction

Mitochondrial dysfunction is a central contributor to COPD-associated exercise intolerance and includes reduced oxidative phosphorylation capacity, altered mitochondrial density, respiratory-chain impairment, increased reactive oxygen species production, and reduced fatigue resistance [2,24,25,30]. MicroRNAs can regulate mitochondrial translation in muscle differentiation models [47], and EV-associated regulation is biologically plausible; however, available COPD evidence supports muscle-level dysfunction rather than a demonstrated lung-to-muscle EV mechanism.

### 5.3. Satellite Cells, Fibro-Adipogenic Progenitors, and the Endothelial Interface

Skeletal muscle homeostasis depends on interactions among myofibres, satellite cells, fibro-adipogenic progenitors, endothelial cells, immune cells, and extracellular matrix [22,28,35,36,48,49,50]. Satellite cells mediate repair, fibro-adipogenic progenitors support regeneration and matrix remodelling but can contribute to fibrosis or adipogenesis when dysregulated, and endothelial cells provide oxygen and paracrine signals that coordinate muscle remodelling.

COPD muscle data indicate disruption of this regenerative niche. Patient-derived satellite cells form smaller myotubes and show increased oxidative stress [28]; the COPD systemic environment impairs myogenic function in vitro, although conditioned medium does not isolate EV-specific effects [31]; satellite cells are located farther from capillaries [22]; and an emphysema model shows impaired myogenesis and altered autophagy [51]. These findings identify myogenic, stromal, and vascular cells as candidate EV recipients and justify EV-depletion/rescue experiments, but they do not prove that lung-derived EVs disrupt the niche.

### 5.4. Hypoxia, Oxygen Desaturation, and EV Signalling

Altered oxygenation may be an important modifier of the lung–muscle EV axis. Patients with COPD or IPF may experience sustained resting hypoxaemia, exertional desaturation, nocturnal hypoxaemia, or short recurrent episodes of reduced oxygen saturation. These patterns are not interchangeable: their duration, depth, frequency, and interaction with inflammation, perfusion, and physical activity may differentially regulate EV biogenesis and cargo. Hypoxia-inducible-factor and redox-sensitive pathways can influence endosomal trafficking, cellular stress responses, endothelial activation, and microRNA expression, creating a plausible mechanism through which oxygen instability could alter both pulmonary and muscle EV signalling.

Direct COPD/IPF lung–muscle evidence is not yet available. In vitro intermittent hypoxia produced cell-selective changes in EV cargo and biological effects [52], and an acute exercise bout under hypoxic conditions altered circulating EV responses and skeletal-muscle multivesicular-body markers in healthy and prediabetic participants [53]. In a lung-cancer-related experimental context, circulating EVs from individuals with COPD transferred HIF-1alpha and modified recipient-cell phenotype [54]. These studies establish that hypoxia-linked EV cargo can be biologically active, but they do not demonstrate that oxygen-sensitive pulmonary EVs reach skeletal muscle or cause wasting in COPD or IPF.

Candidate oxygen-responsive EV signals for prospective study include miR-210, HIF-associated proteins, glycolytic enzymes, VEGF-related or endothelial-activation proteins, adhesion molecules, and oxidative or inflammatory mediators. At present, these should be described as hypothesis-generating cargo rather than validated biomarkers of lung–muscle communication. Future studies should combine serial EV sampling with contemporaneous arterial oxygenation and pulse-oximetry phenotyping at rest, during standardised exertion, and during sleep; source-resolved BAL, plasma, and muscle-interstitial analyses would help separate disease severity from the specific effect of oxygen desaturation.

## 6. Exercise Intolerance and the Exercise Paradox

Exercise intolerance in COPD and IPF is multifactorial. Ventilatory limitation, dynamic hyperinflation, hypoxaemia, pulmonary vascular disease, cardiac comorbidity, deconditioning, anaemia, systemic inflammation, mitochondrial dysfunction, and skeletal muscle wasting may all contribute [2,26,27,29,30]. EV biology should therefore be integrated into a multisystem model rather than presented as a single dominant mechanism. Exercise-derived EVs may participate in adaptive inter-organ signalling, but their role in chronic respiratory disease-associated muscle dysfunction remains incompletely defined [55,56,57].

The exercise paradox in COPD is context-dependent. Pulmonary rehabilitation improves functional outcomes and may improve endothelial function [58]. Acute exercise in severe COPD may nevertheless provoke heterogeneous oxidative stress responses, particularly in patients with severe hypoxaemia, low antioxidant reserve, endothelial dysfunction, or advanced muscle oxidative impairment [26,27,58]. Endothelial-derived EVs may increase after acute exercise, but this signal may reflect vascular stress, adaptive communication, or both [37].

Reactive oxygen species may be adaptive at physiological levels and injurious when excessive or sustained [26,27]. A xanthine oxidase-reactive oxygen species-endothelial EV pathway is therefore plausible, but the degree to which it causes muscle wasting remains unknown. This uncertainty supports individualised, supervised rehabilitation rather than avoidance of exercise.

## 7. Therapeutic EV Strategies

Therapeutic design should be considered before individual examples. Mesenchymal stromal cell-derived extracellular vesicles (MSC-EVs) and other therapeutic EV platforms depend on donor-cell source, manufacturing conditions, vesicle composition, surface molecules, cargo loading, route of administration, biodistribution, clearance, and recipient-cell uptake. Engineering may modify tetraspanins, integrins, targeting peptides, membrane properties, or RNA/protein cargo to improve stability and tissue delivery, but every modification requires potency, off-target, immunogenicity, and safety assessment [38,43].

Preclinical muscle studies provide proof of mechanism outside COPD and IPF. Bone marrow-derived mesenchymal stromal cell exosomes attenuated dexamethasone-induced myotube and mouse muscle atrophy through the miR-486-5p/FoxO1 axis [59]. Tonsil-derived mesenchymal stromal cell exosomes reduced activin A-associated muscle loss through miR-145-5p-mediated regulation of activin receptors in cell and chemotherapy-conditioning models [60]. A CD63-anchored myostatin propeptide system improved outcomes in mdx mice, while exosome mimetics and magnetic targeting have been explored in bone or dystrophic-muscle models [61,62,63]. These studies demonstrate anti-catabolic or delivery feasibility, not efficacy in respiratory disease-associated muscle wasting.

Translation in pulmonary disease has begun but remains disease- and endpoint-specific. A 2025 study reported nebulised human umbilical cord mesenchymal stromal cell-derived EVs in experimental pulmonary fibrosis and a small clinical cohort, providing early evidence of pulmonary delivery, safety, and feasibility [64]. Inhalable dry-powder formulations also address stability and local delivery [65]. Neither line of evidence evaluated skeletal muscle uptake, muscle mass, strength, mitochondrial function, or exercise-mediated lung–muscle signalling; therefore, they should not be interpreted as treatment evidence for COPD- or IPF-associated muscle dysfunction.

Major barriers include reproducible clinical-grade production, donor variability, batch consistency, validated potency assays, dose and schedule, route-dependent biodistribution, off-target uptake, immune compatibility, long-term safety, storage stability, and scalability [38,64,66,67,68]. Lung–muscle applications also require a clear therapeutic target: the injured lung, the systemic inflammatory/vascular compartment, or skeletal muscle and its regenerative niche. Current evidence supports early translation for selected pulmonary indications and preclinical rationale for muscle applications, not clinical readiness for the lung–muscle axis.

Source-dependent cargo and oxygen-sensitive EV biology also have therapeutic implications. Lung-targeted EV strategies would ideally suppress epithelial, endothelial, immune, or fibroblast injury, whereas muscle-targeted platforms would require anti-catabolic, mitochondrial, vascular, or regenerative potency in myofibres and their niche. Hypoxic preconditioning of bone-marrow mesenchymal stromal cells has enriched exosomal miR-210-3p and promoted satellite-cell activation and skeletal-muscle regeneration through the KLF7 pathway in a preclinical model [39]. This provides a regenerative proof of concept, but oxygen conditioning is potentially double-edged because hypoxia may also enrich pathogenic inflammatory or profibrotic cargo. Oxygen tension should therefore be treated as a controlled manufacturing variable, with predefined cargo, potency, biodistribution, and safety testing. No study has established the efficacy of such an approach for COPD- or IPF-associated muscle dysfunction.

## 8. Evidence Map and Evidence Hierarchy

**Figure 1:**
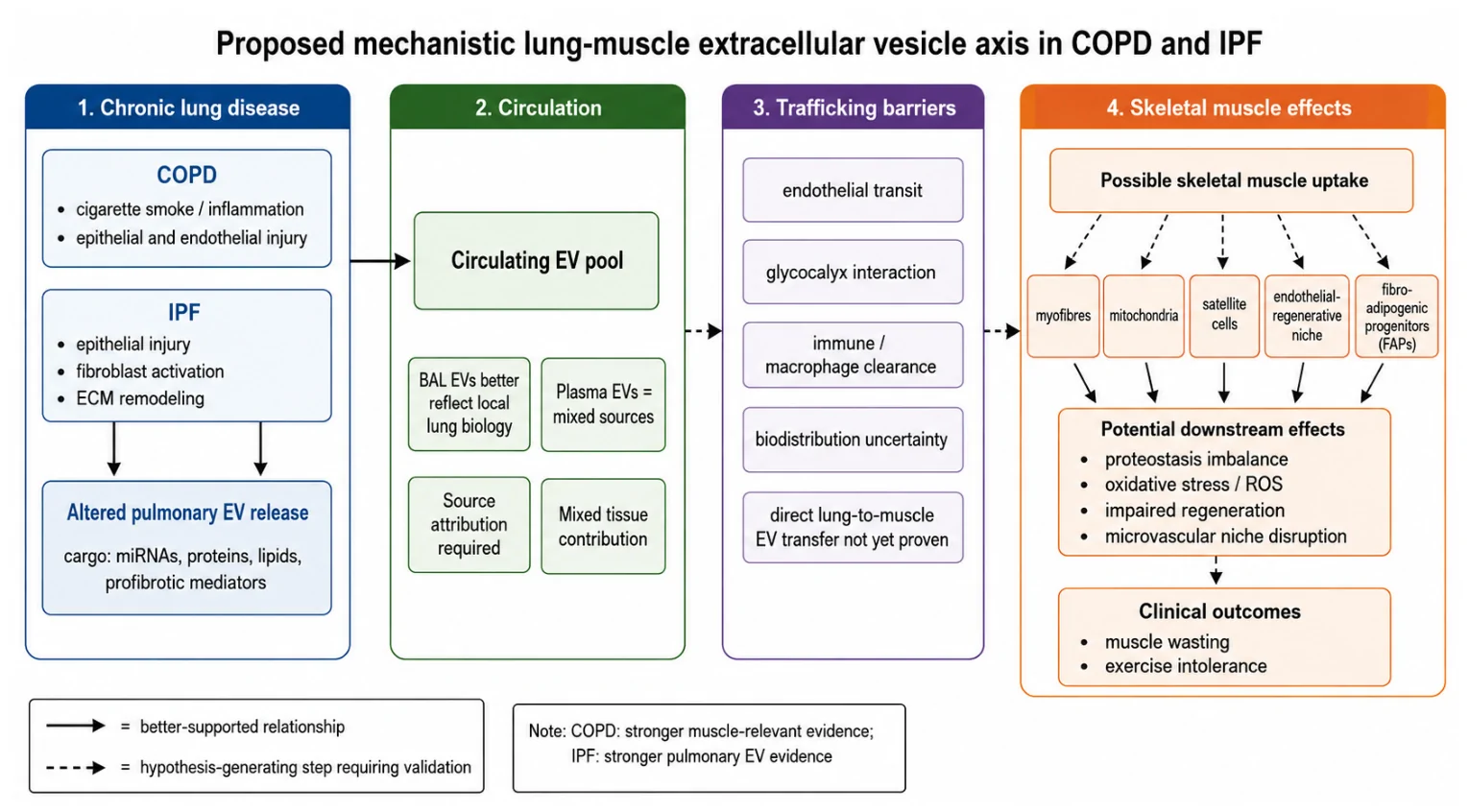
Proposed lung–muscle extracellular vesicle axis in COPD and IPF. Chronic lung injury may alter pulmonary extracellular vesicle (EV) release and cargo. After entering the circulation, EVs face endothelial, immune-clearance, and biodistribution barriers before potentially reaching skeletal muscle. Dashed arrows indicate unproven, hypothesis-generating steps. Evidence is stronger for muscle abnormalities in COPD and for intrapulmonary EV biology in IPF. Abbreviations: BAL, bronchoalveolar lavage; COPD, chronic obstructive pulmonary disease; ECM, extracellular matrix; EVs, extracellular vesicles; FAPs, fibro-adipogenic progenitors; IPF, idiopathic pulmonary fibrosis; miRNAs, microRNAs; ROS, reactive oxygen species.

Figure 1 and Table 1 and Table 2 summarise the evidence-weighted framework. Direct human COPD muscle findings, intrapulmonary COPD/IPF EV biology, vascular or trafficking precedents, and therapeutic studies are deliberately separated because they answer different questions and should not be assigned equivalent causal weight.

## 9. Discussion

The lung–muscle EV axis is a coherent conceptual model, but current evidence supports a graded rather than definitive interpretation. COPD provides direct muscle-relevant evidence for miR-542 dysregulation, impaired proteostasis, mitochondrial dysfunction, oxidative stress, and regenerative-niche abnormalities [13,14,15,22,24,25,28,29,30,31,35,47,51]. These findings define biological targets and recipient-cell phenotypes; they do not prove that pathogenic cargo is released by the lung and delivered by EVs in vivo.

IPF requires a different evidentiary framing. Its strongest data concern EV-mediated pulmonary epithelial reprogramming, fibroblast signalling, and fibrotic remodelling [10,16,17,18]. The presence of systemic muscle weakness or low muscle mass in IPF does not establish an EV mechanism because hypoxaemia, inactivity, nutritional vulnerability, inflammation, and disease severity provide alternative explanations [4,5].

The unresolved trafficking barrier is therefore central. EV source attribution in plasma is difficult, and vascular transit, glycocalyx interactions, phagocytic clearance, organ tropism, and recipient-cell specificity may substantially limit delivery to skeletal muscle. Without lineage tracing or validated source-specific labelling, circulating cargo cannot be confidently assigned to pulmonary origin; without uptake and rescue experiments, association cannot be converted into functional causality.

The comparison between muscle- and lung-derived EVs reinforces this limitation. Available studies do not reveal a dependable organ-specific morphology: particle dimensions and ultrastructural appearance overlap, whereas cargo and source-associated markers vary more clearly with cell type and physiological state [8,17,20,21,34,40,41]. SGCA, myomiRs, contractile or metabolic proteins may support a muscle-associated phenotype, while epithelial, inflammatory, endothelial, and profibrotic cargo may support pulmonary attribution. Nevertheless, these are probabilistic signatures, not definitive lineage labels, and their performance has not been validated across COPD or IPF severity stages.

Oxygenation should be incorporated into this prospective framework rather than treated merely as a surrogate of global disease severity. Resting hypoxaemia, exertional or nocturnal desaturation, and brief recurrent hypoxic episodes may produce different HIF- and redox-sensitive EV responses [52,53,54]. Candidate signals include miR-210, HIF-associated proteins, metabolic enzymes, and endothelial-activation mediators, but their pulmonary source, skeletal muscle uptake, and functional relevance remain unverified. Prospective studies should therefore model oxygen exposure quantitatively and temporally and should distinguish stable disease from exacerbation, rehabilitation, and progressive fibrotic states.

Exercise evidence also requires balance. Pulmonary rehabilitation remains beneficial, whereas acute oxidative and endothelial responses vary with disease severity and physiological reserve [26,27,37,58]. Exercise-related EV changes should be interpreted as biomarkers or candidate signals until their source, cargo, recipient cells, and functional effects are demonstrated.

Therapeutic translation is no longer exclusively preclinical for pulmonary fibrosis, because early human feasibility data for nebulised MSC-EVs have emerged [64]. Nevertheless, no therapeutic EV study has established prevention or reversal of COPD- or IPF-associated skeletal muscle dysfunction. This distinction between pulmonary delivery feasibility and lung–muscle efficacy should guide both interpretation and future trial design.

**Figure 2:**
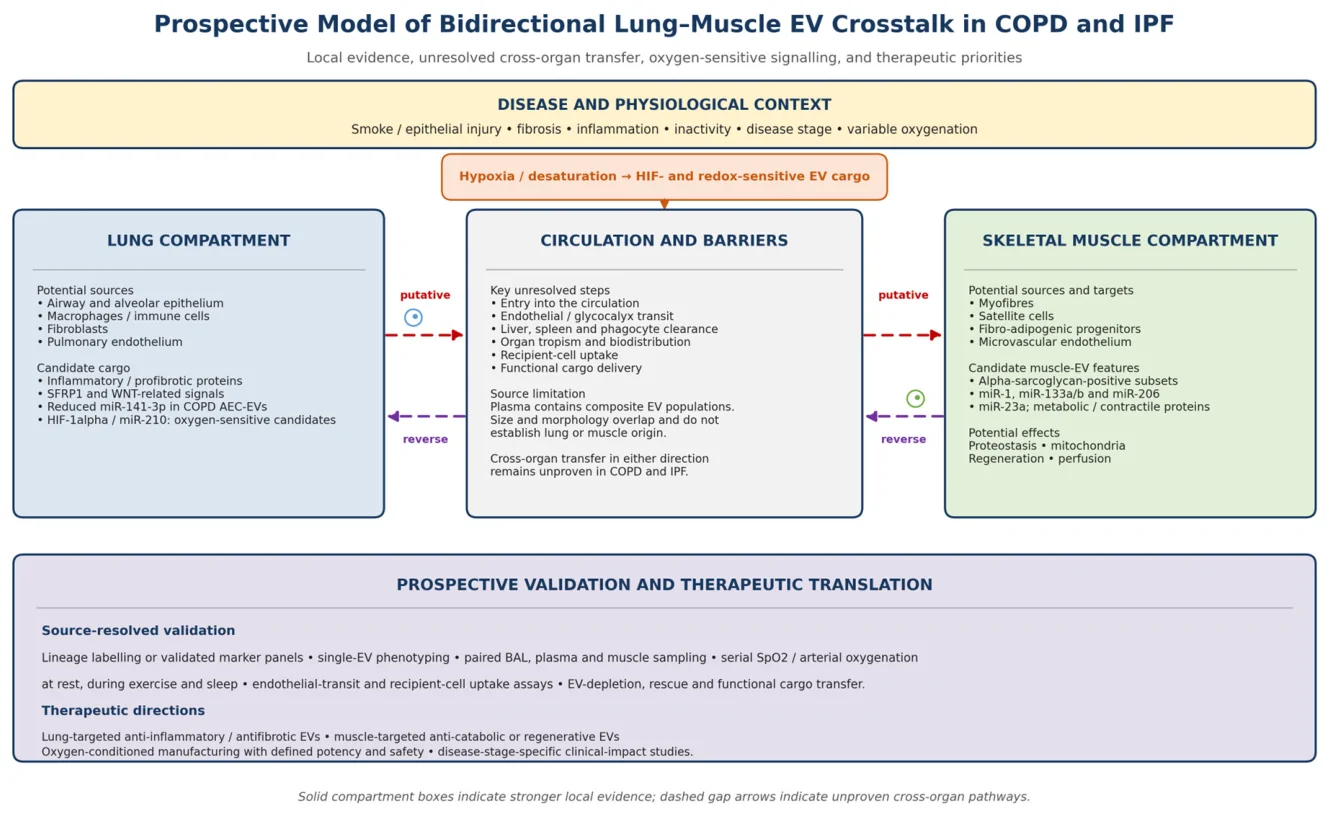
Prospective model of bidirectional lung–muscle extracellular vesicle crosstalk in COPD and IPF. Pulmonary injury and altered oxygenation may change EV release and cargo from epithelial, immune, fibroblast, and endothelial cells. Putative lung-to-muscle transfer must overcome vascular, glycocalyx, clearance, biodistribution, uptake, and bioactivity barriers before influencing myofibres or regenerative-niche cells. Skeletal muscle may release SGCA-positive EV subpopulations, myomiRs, and metabolic or contractile proteins during contraction, disuse, or atrophy, permitting a potential reverse muscle-to-lung or systemic signal. Solid compartment boxes indicate comparatively stronger local evidence; dashed arrows represent unproven cross-organ pathways. The lower panel identifies priorities for source-resolved validation and therapeutic translation. Abbreviations: AEC, airway epithelial cells; BAL, bronchoalveolar lavage; COPD, chronic obstructive pulmonary disease; EVs, extracellular vesicles; fibro-adipogenic progenitors; HIF, hypoxia-inducible factor; IPF, idiopathic pulmonary fibrosis; microRNAs; alpha-sarcoglycan; SpO2, peripheral oxygen saturation.

Figure 2 integrates the current evidence into a prospective bidirectional model. It separates comparatively stronger local pulmonary and muscle EV biology from the unproven vascular transit and functional cargo-transfer steps, while positioning oxygenation, disease stage, and therapeutic engineering as testable modifiers.

## 10. Materials and Methods

This narrative review synthesises evidence relevant to EV-mediated inter-organ communication, chronic lung disease-associated skeletal muscle dysfunction, exercise intolerance, endothelial biology, and emerging EV-based therapeutic platforms. It was designed as a mechanistically structured and evidence-weighted synthesis rather than a systematic review or meta-analysis.

Targeted PubMed searches were performed and last updated on 15 June 2026. Search combinations included extracellular vesicles, small extracellular vesicles, exosomes, microvesicles, COPD, chronic obstructive pulmonary disease, idiopathic pulmonary fibrosis, pulmonary fibrosis, skeletal muscle wasting, sarcopenia, cachexia, exercise intolerance, miR-542, miR-23a, satellite cells, fibro-adipogenic progenitors, endothelial extracellular vesicles, mesenchymal stromal cell extracellular vesicles, inhalable extracellular vesicles, and engineered exosomes. Priority was given to human and translational studies, systematic reviews, mechanistic investigations directly relevant to EV biology, and recent primary studies. A focused update addressing source-dependent EV morphology and cargo, hypoxia, oxygen desaturation, HIF signalling, miR-210, myomiRs, alpha-sarcoglycan, and therapeutic oxygen conditioning was performed on 22 July 2026.

Studies were considered central when they addressed EV biology in COPD or IPF; skeletal muscle dysfunction in chronic respiratory disease; EV cargo affecting muscle, endothelial, immune, or regenerative-niche function; exercise-related EV responses; or therapeutic EV platforms relevant to lung or muscle. Evidence was appraised thematically according to whether it was direct human muscle evidence, pulmonary EV evidence, indirect systemic or vascular evidence, or preclinical/clinical therapeutic evidence. Because the search was targeted and narrative, the review does not claim exhaustive study capture or permit quantitative risk-of-bias assessment.

## 11. Conclusions

EV-mediated inter-organ communication offers a plausible framework for studying skeletal muscle dysfunction and exercise intolerance in chronic respiratory disease. COPD currently provides the strongest direct muscle-relevant evidence, while IPF provides stronger intrapulmonary EV evidence. miR-542-3p/-5p and miR-23a are informative muscle-related signals, but neither should be described as proven lung-derived EV cargo affecting skeletal muscle.

The decisive next step is not additional association alone. Studies must identify EV source, demonstrate vascular transit and recipient-cell uptake, and show functional cargo activity using source-labelled systems, EV-depletion and rescue, human-relevant endothelial barriers, single-vesicle phenotyping, and integrated outcomes across myofibres, mitochondria, satellite cells, fibro-adipogenic progenitors, and the microvasculature. Until those requirements are met, EVs should be described as candidate mediators and emerging therapeutic platforms—not established drivers or clinically validated treatments of respiratory disease-associated muscle wasting.

Neither conventional EV size nor morphology currently distinguishes pulmonary from skeletal muscle origin. Molecular cargo and source-associated marker panels are more informative but remain imperfect, and no paired source-resolved human study has validated them across COPD or IPF severity. Prospective work should therefore combine lineage-aware EV phenotyping with contemporaneous resting, exertional, and nocturnal oxygenation data and functional transfer experiments.

## Figures and Tables

**Table 1:** Evidence map of studies relevant to the lung–muscle EV axis.

Study	Model/EV Source	Key Finding	Interpretive Value
Gomez et al., 2022 [9]	COPD systematic review; mixed pulmonary and circulating EVs	COPD EV studies are promising but methodologically heterogeneous.	Supports COPD EV relevance while highlighting characterisation and source-attribution limits.
Farre-Garros et al., 2019 [14]	Human COPD quadriceps; muscle tissue and mechanistic myotube assays	miR-542-3p/-5p are elevated and linked to impaired protein synthesis.	Direct COPD muscle evidence; not proof of lung-derived EV transfer.
Betz et al., 2024 [22]	Human COPD muscle biopsy and niche analysis	Satellite cells are located farther from capillaries in COPD muscle.	Supports disruption of the endothelial-satellite cell interface.
Burgy et al., 2024 [17]	Pulmonary fibrosis; fibroblast-derived EVs	Fibroblast EVs contain SFRP1 and mediate fibrosis-related signalling.	Strong pulmonary EV evidence; muscle implications remain indirect.
Tine et al., 2023 [19]	Human COPD BAL and plasma EVs	Circulating EVs did not simply mirror BAL EV profiles.	Highlights compartment and source-attribution limitations.
Bartel et al., 2024 [20]	Human COPD BAL EVs	Altered EV protein and microRNA signatures were identified in BAL fluid.	Supports local pulmonary EV alteration, not cross-organ transfer.
Hoshino et al., 2015 [42]; Kang et al., 2021 [43]	Cancer organotropism and animal biodistribution studies	Surface integrins may affect tropism; administered EVs often accumulate in liver, spleen, lung, and phagocytic compartments.	Biological precedents only; not COPD/IPF lung–muscle evidence.
Nieri et al., 2020 [37]	COPD acute exercise pilot; endothelial cell-derived EVs	Acute exercise altered endothelial EV profiles.	Supports a vascular EV response; functional muscle effects are unknown.
Li et al., 2021 [59]; Cho et al., 2021 [60]	Non-COPD muscle atrophy models; mesenchymal stromal cell (MSC)-derived exosomes	Experimental muscle atrophy was attenuated through miR-486-5p/FoxO1 and miR-145-5p/activin pathways.	Anti-atrophy proof of mechanism, not respiratory disease efficacy.
Ran et al., 2020 [61]; Villa et al., 2024 [63]	Engineered or targeted exosome systems in muscle disease models	Targeted platforms improved outcomes in experimental dystrophic muscle.	Delivery proof of concept; not COPD/IPF-ready therapy.
Li et al., 2025 [64]	Nebulised human umbilical cord MSC-EVs (hUCMSC-EVs); pulmonary fibrosis models and small clinical cohort	Early pulmonary delivery, safety, and feasibility data were reported.	Clinical pulmonary feasibility; no skeletal muscle or lung–muscle endpoints.
Guescini et al., 2015 [40]; Watanabe et al., 2022 [41]	Muscle-associated plasma EVs and experimental skeletal muscle EVs	SGCA, myomiRs, and distinct cargo were associated with muscle EVs; many experimental EVs remained in the muscle interstitium.	Candidate source signatures; neither morphology nor a single marker is tissue-exclusive.
Aas et al., 2023 [21]	Human myotube EVs after electrical-pulse stimulation	Stimulation changed miRNA and protein cargo without changing EV size or number.	Supports cargo differences rather than a unique muscle-EV morphology.
Wang et al., 2026 [34]	Primary COPD airway epithelial cell-derived EVs	Reduced EV miR-141-3p promoted endothelial inflammation and atherosclerotic change through PDCD4/NF-kappaB signalling.	Direct pulmonary source and vascular activity; not evidence of muscle uptake.
Sanz-Rubio et al., 2021 [52]; Warnier et al., 2022 [53]	Intermittent-hypoxia models and acute exercise under hypoxia	Hypoxia modified EV cargo or release in a context-dependent manner.	Candidate oxygenation modifier; indirect for COPD/IPF transfer.

Note: Claims requiring lung-origin source attribution, endothelial transit, skeletal muscle uptake, and functional cargo activity remain hypothesis-generating until directly demonstrated. Abbreviations: BAL, bronchoalveolar lavage; MSC, mesenchymal stromal cell; hUCMSC-EVs, human umbilical cord mesenchymal stromal cell-derived extracellular vesicles.

**Table 2:** Evidence hierarchy for principal claims.

Claim Category	Supported Claim	Evidence Level
Direct evidence	miR-542-3p/-5p are elevated in COPD quadriceps and affect protein-synthesis pathways [14].	Human COPD muscle plus mechanistic assays.
Direct evidence	COPD muscle shows impaired satellite-cell function and altered capillary proximity [22,28,31].	Human biopsy and cell data.
Indirect evidence	COPD pulmonary and circulating EV populations/cargo are altered [9,11,12,19,20,33,34].	Human EV profiling and reviews; source attribution remains limited.
Indirect evidence	IPF EVs participate in profibrotic pulmonary signalling [10,16,17,18].	Pulmonary fibrosis studies and reviews.
Hypothesis-generating	Lung-derived EVs cross endothelium and deliver functional cargo to skeletal muscle.	No integrated human source-tracing and functional-transfer evidence.
Early pulmonary translation	Nebulised MSC-EVs have early pulmonary-fibrosis feasibility data [64].	Small clinical cohort; no muscle outcomes.
Hypothesis-generating therapy	EV platforms show preclinical therapeutic potential, but have not been demonstrated to treat COPD- or IPF-associated skeletal muscle dysfunction [59,60,61,62,63,64,65,66,67,68].	No disease-specific clinical efficacy data for muscle wasting.
Comparative morphology	No validated size or morphology separates lung- from muscle-derived EVs; cargo and marker panels are more informative [8,21,34,40,41].	Method-dependent evidence; no paired source-resolved COPD/IPF comparison.
Hypoxia-modified signalling	Hypoxia/desaturation may alter EV release and HIF-linked or miR-210 cargo [39,52,53,54].	Plausible, but no integrated COPD/IPF source-tracing and muscle-functional evidence.

## Data Availability

No new data were created or analysed in this study.
